# Determining the relationship between over-care burden and coping styles, and resilience in mothers of children with autism spectrum disorder

**DOI:** 10.1186/s13052-023-01465-0

**Published:** 2023-05-08

**Authors:** Shabnam Rasoulpoor, Nader Salari, Amir Shiani, Behnam Khaledi-Paveh, Masoud Mohammadi

**Affiliations:** 1 Department of Psychiatric Nursing, School of Nursing and Midwifery, Urmia University of Medical Sciences, Urmia, Iran; 2 Department of Biostatistics, School of Health, Kermanshah University of Medical Sciences, Kermanshah, Iran; 3 Department of Radiology and Nuclear Medicine, School of Allied Medical Sciences, Kermanshah University of Medical Sciences, Kermanshah, Iran; 4 Sleep Disorders Research Center, Kermanshah University of Medical Sciences, Kermanshah, Iran; 5 Cellular and Molecular Research Center, Gerash University of Medical Sciences, Gerash, Iran

**Keywords:** Autism spectrum disorder, Care burden, Coping style, Resilience

## Abstract

**Background:**

Stress and over-care burden are problems for mothers of children with Autism Spectrum Disorder (ASD). Therefore, it seems necessary to evaluation of coping with stress according to the burden of care for these mothers. This study aimed to determine the relationship between care burden with coping styles and resilience of mothers of children with ASD.

**Methods:**

The present study is a descriptive-analytical study performed on mothers of children with ASD in Kermanshah, Iran. Participants in the study were selected by convenience sampling. A Demographic questionnaire, Caregiver Burden Inventory (CBI), Connor-Davidson Resilience Scale (CD-RISC), and Coping strategies questionnaire (CSQ) were used for collecting data. Then it was analyzed through an independent t-test, ANOVA, and Pearson correlation test.

**Results:**

The mean total score of the burden of care was 95.5 ± 9.1, resilience was 52.7 ± 8.7, and coping styles were 92.4 ± 8.4. Mothers of children with autism experience a severe burden of care and moderate levels of resiliency. There was also a significant negative correlation between the burden of care and resilience) p < 0.001, r = -0.536), but no significant correlation was found between burden of care and coping style) p = 0.937, r = -0.010).

**Conclusion:**

According to the results of this study, it is necessary to pay more attention to the factors affecting resiliency. Also, considering the significant relationship between burden of care and resiliency, teaching strategies to increase resiliency can be used in the educational program for mothers with autistic children.

**Supplementary Information:**

The online version contains supplementary material available at 10.1186/s13052-023-01465-0.

## Background

Autism spectrum disorder is a disease or a set of diseases related to the development of the nervous system, which is characterized by impaired social interaction and communication and limited and repetitive behaviors [1]. The prevalence of autism has been continuously increasing over the past two decades [2]. According to the Centers for the Disease Control and Prevention (CDC) report in the United States, the prevalence is estimated to be one in every 59 people in 2014 and one in every 54 people in 2016 in 8-year-old children [3].

Inability to take care of oneself and dependence on caregivers are the most critical problems of children with autism [4]. Caring for children with autism spectrum disorder is a stressful process [5]. The stress caused by raising a sick or disabled child creates a burden of care [6]. The burden of care is defined as the physical, psychological, social, or economic reactions that appear in the caregiver during care, and it is defined as stress or negative experiences caused by care in the caregiver. Various studies on the relationship between maternal stress, Discordant behavior, and low social support have been associated with an increased burden of care [7, 8].

The burden of care imposed on mothers of children with autism leads them to find strategies to deal with caregiving stress [9]. Coping styles have been proposed as a mechanism by which people respond to threats caused by stress [10]. Coping styles are divided into two general types, problem-focused coping (strategies aimed at solving the problem or doing something to change the source of stress) and emotion-focused coping (strategies aimed at reducing or managing feelings of distress associated with the stressor) [11, 12]. In studies of parents of individuals with autism, the use of emotion-focused coping styles (e.g., denial and avoidance) has generally been associated with higher levels of psychological distress. In comparison, the use of problem-focused coping styles (for example, planning and taking action to deal with this problem) has often been associated with improved mental health outcomes [11]. According to studies, the stress of parents of autistic children affects the care burden [11–13]. Also, effective coping styles against stress reduce the incidence of depression and other mental disorders in these parents [14].

Researchers believe there are moderating factors between parents’ mental pressures and child disorders. The presence of some characteristics can increase the internal resistance to mental stresses. It is one of the factors that moderates resilience [15]. Resilience is returning to the initial equilibrium or reaching higher stability in a threatening situation [16]. People with a high-level resilience are likely to deal with their exceptional children logically and correctly [17]. Several studies’ results have indicated a significant relationship between resilience and care burden in caregivers of patients [18, 19]. Based on the results, high resilience in caregivers may help prevent caregiving burden, reduce its severity, and facilitate effective coping styles [19].

Therefore, considering the increasing prevalence of autism, the importance of women’s position in society and family, and their essential role in caring for children with autism spectrum disorder, we decided to design and implement this study. This study aimed to investigate the relationship between caregiving burden and coping styles and resilience in mothers of autistic children. Also, the research hypothesis was that there is a relationship between the burden of care and coping methods and resilience in mothers of autistic children.

## Methods

### Study design and setting

This study was conducted using a descriptive-analytical research plan between July and December 2021 at the Autism Center affiliated with Kermanshah University of Medical Sciences. The following two objectives were investigated in the study: “Assessment of caregiving burden, coping styles and resilience of mothers of autistic children” and “Relationship between caregiving burden and coping styles and resilience in mothers of autistic children.“

### Participants and sampling

For sampling, after obtaining ethics approval, the researcher went to the Autism Center affiliated with Kermanshah University of Medical Sciences and received the phone numbers of the mothers of autistic children who met the inclusion criteria. Then researcher contacted mothers with autistic children, and mothers willing to participate in this study were invited. Therefore, 80 mothers with autistic children were selected to participate in the research using the available sampling method. It should be noted that their families and children visited the center one day a week to receive special educational and counseling services. The questionnaires were delivered to the volunteer mothers on the same day, and they were asked to complete the questionnaires if they agreed to participate in the study. Completion of the forms and tools took approximately 40 min.

The criteria for entering the study were as follows: having at least one child aged 3 to 15 with a definite diagnosis of autism, the absence of other physical, cognitive or mental disorders in the mother, and the willingness and consent of the mother to participate in the study. Participants who could not answer more than half of the questions in their questionnaires or did not return their questionnaires were excluded. The majority of questionnaires (87%) were collected in November, and finally, 69 mothers completed the questionnaires. One of the main reasons for mothers not participating in this study was containing many questions in the questionnaire and not having enough time to complete the questionnaire.

## Questionnaire

### Demographic information questionnaire

Demographic information included the child’s age, mother’s age, child’s gender, economic status, number of children, mother’s education level, mother’s status, marital status, and age of autism diagnosis.

### Caregiver burden inventory (CBI)

The caregiving burden questionnaire was created in 1989 by Novak and Guest to measure the objective and subjective burden of care. This questionnaire has 24 questions and five subscales, which are: time-dependent burden of care, developmental burden of care, physical burden of care, social burden of care, and emotional burden of care. This questionnaire is based on a 5-point Likert scale from completely disagree [1] to completely agree [5]. Based on this, the scores of this questionnaire ranged from 24 to 120, where scores of 24 to 47 are considered mild care pressure, 48 to 71 as moderate care pressure, 72 to 95 as severe care pressure, and 96 to 120 as very severe care pressure. In the study of Novak et al., the validity and reliability of the care burden questionnaire were measured in caregivers, and Cronbach’s alpha for different subscales was obtained from 73 to 87% [20]. This tool has been translated into different languages and used in various countries, including; Brazil, Turkey, China, and Italy, and it has a high level of validity and reliability [21–23].

### Connor-Davidson resilience scale (CD-RISC)

Connor and Davidson prepared the resilience scale in 2003 to measure the power to deal with pressure and threat. This questionnaire has 25 questions and five subscales: perception of personal competence, correspondence to trust in one’s instincts, tolerance of negative affect, positive acceptance of change, secure relationships, control, and spiritual influences. For each question, a five-choice grading scale is considered, scored from zero (completely false) to four (always true). The scoring method of this questionnaire is 0-100. Scores of 0–50 indicate low resilience, 50–75 mean resilience, and 75 and above show high resilience. Connor and Davidson reported the Cronbach’s alpha coefficient for the resilience scale, 0.89 [24].

### Coping strategies questionnaire (CSQ)

Lazarus and Folkman designed the Coping Strategies Questionnaire in 1980. It is a tool to study how people cope with stress. This questionnaire has 66 questions and eight subscales. The problem-based coping part includes the four characteristics of seeking social support, responsibility, managerial problem-solving, and positive re-evaluation. The emotion-based coping part consists of the four attributes of direct confrontation, escape and avoidance, avoidance, and self-control. The questions are scored on a 4-point Likert scale (from 0: I have not used it at all to 3: I use it a lot). In this questionnaire, if the calculated score is between 0 and 66, it is a sign of using a low coping style in the person, and if the calculated score is between 66 and 110, it is a sign of using an average coping style. Finally, if the calculated score is 110 or more, it is a sign of using a high coping style in the person. Lazarus reported internal consistency of 0.79 to 0.66 for each coping style [25]. Rezakhani et al. obtained the reliability coefficient of this tool using Cronbach’s alpha method of 0.80, and the research results showed that this questionnaire has acceptable validity and reliability [26].

### Statistical methods

Data analysis was done using SPSS version 25 software. Descriptive statistics methods (including setting frequency distribution tables, calculating numerical indices such as mean, the standard deviation for quantitative traits, and percentage for qualitative traits) were used to summarize and describe the variables. Kolmogorov-Smirnov test was used to check the normality of the distribution of the scores of the investigated indicators. Also, an independent t-test was used for two-category variables, and a one-factor analysis of variance was used for three-category and higher demographic variables at a significance level of less than 0.05. Pearson’s correlation test was used to investigate the correlation between the burden of care, coping styles, and resilience of mothers of children with autism.

### Ethics approval and consent to participate

The Ethics Committee approved the study of Kermanshah University of Medical Sciences (IR.KUMS.REC.1400.040). Also, at the beginning of the study, the researcher introduced herself to the research units, explained the way of conducting the research and the purpose of its implementation, and assured them that all information would remain confidential and the participants could withdraw from the study at any time. To gain the trust of the research units, we refrained from asking and including their names, family names, and addresses. Finally, after providing sufficient information about the study, written informed consent was obtained from all participants.

## Results

### Demographic information

Of the 69 mothers who participated in the study, the participants’ age range was between 26 and 54 years, with an average of 38.4 ± 7 years. Most participants (94.2%) were married, 50.7% of whom had one child. The income of more than half of the mothers (78.2%) was equal to the expenses. Also, 56.5% had a university education, 30.4% were employed, and 79.7% had a son with autism. The age range of children with autism was between 3 and 15 years, with an average of 9.4 ± 3.8 years. The average age of autism diagnosis in children was 3.3 ± 1. The minimum and maximum age of diagnosis were 2 and 5 years, respectively (Table 1).

### The relationship between caring burden and demographic information

This study’s findings showed a significant relationship between the burden of care and the mother’s age, number of children, mother’s employment status, child’s gender, and economic status. So, mothers with more children, low economic status, and a daughter with autism reported more burden of care (Table 1).

**Table 1:** Participants’ demographic characteristics and caring burden scores

Demographic variables	Number	Care burden Means ± SD	p. value
Mother’s age (Year)			
23–35 years	43	93.67 ± 8.68	P = 0.024
36 years and over	26	98.73 ± 9.07	
**Mother’s educations**			
Primary education	2	98.00 ± 12.73	P = 0.880
High school	28	96.21 ± 9.37	
University	39	95.21 ± 9.00	
**Marital status**			
Married	65	95.97 ± 8.67	P = 0.153
Divorce	4	89.25 ± 14.80	
**Employment status**			
Employed	21	92.24 ± 9.70	P = 0.043
Unemployed	48	97.04 ± 8.53	
**Income status**			
The income is less than expenses	6	100.33 ± 8.36	P = 0.046
The income is equal to the expenses	54	96.09 ± 8.40	
The income is more than expenses	9	89.33 ± 11.46	
**Number of children**			
**1**	35	92.54 ± 9.86	P = 0.017
2	31	98.71 ± 7.08	
3 and more	3	98.67 ± 9.29	
**Sex of children**			
Girl	14	101.79 ± 6.68	P = 0.004
Boy	55	94.00 ± 9.01	
**Children’s age**			
3–6	21	98.05 ± 9.18	P = 0.277
7–10	17	93.41 ± 10.98	
11–15	31	95.10 ± 7.76	
**The child’s age at diagnosis First year of the life**			
2	11	91.91 ± 8.30	P = 0.277
3	32	97.84 ± 10.10	
4 years and over	26	94.35 ± 7.57	

### Caring burden, coping styles and resilience in the participants

The average burden of care of mothers participating in the present study was 95.5 ± 9.1, which shows that the care load is severe. The average resilience score was 52.7 ± 8.7, which indicates an average level of resilience. Also, the average score of coping styles was 92.4 ± 8.4, indicating an average level of coping style (Tables 2 and 3).

**Table 2:** Means and standard deviations of the participants’ caring burden, coping styles and resiliency scores

Variable	Dimension	Means ± SD Per dimension	Total Means ± SD
caring burden	Time-Dependence Burden	20.3 ± 2.6	95.5 ± 9.1
	Developmental Burden	20.2 ± 2.9	
	Physical Burden	15.8 ± 2.3	
	Social Burden	19.4 ± 3.1	
	Emotional Burden	19.7 ± 3.3	
Resilience	personal competence	17.01 ± 3.4	52.7 ± 8.7
	corresponds to trust in one’s instincts and tolerance of negative affect	14.5 ± 0.2	
	positive acceptance of change, and secure relationships	10.6 ± 2.9	
	Control	6.1 ± 1.9	
	Spiritual influences	4.4 ± 1.4	
coping styles	seeking social support	11 ± 2.3	92.4 ± 8.4
	Responsibility	7.1 ± 2.4	
	problem-solving	11.3 ± 1.7	
	positive re-evaluation	13.2 ± 2.5	
	problem-based coping	42.6 ± 2.7	
	direct confrontation	11.5 ± 2.5	
	Avoidance	11.3 ± 2.4	
	escape and avoidance	14.8 ± 3.1	
	self-control	12.1 ± 2.7	
	emotion-based coping	49.7 ± 7.09	

**Table 3:** Frequency of caring burden, coping styles and resilience of the participants

Variable		Number (%)
caring burden	Low	0(0)
	Medium	2(2.9)
	Severe	26(37.7)
	Very severe	41(59.4)
Resilience	Low	25(36.2)
	Medium	44(63.8)
	High	0(0)
coping styles	Low	0(0)
	Medium	10(14.5)
	High	59(85.5)

### The relationship between caring burden, coping styles and resilience in the participants

This study’s findings showed an inverse relationship between caregiving burden and resilience in mothers of children with autism (p < 0.001, r = -0.536). Still, there was no significant relationship between caregiving burden and coping strategies. (0.937, r = 0.010) (Table 4).

**Table 4:** The relationship between caring burden, coping styles and resilience

Variable	caring burden	R	P- value
Pearson Correlation	Resilience	-0.536	P < 0.001
	coping styles	-0.010	0.937

## Discussion

This study showed that mothers of children with autism reported higher caregiving burdens, moderate levels of resilience, and moderate levels of coping styles. There is an indirect correlation between caregiving burden and resilience, but no significant relationship was found between caregiving burden and coping style.

The results of this study showed that the mother’s age, number of children, employment status, child’s gender, and economic status affect the burden of care imposed on these mothers. The results of previous studies have shown that parents of children with autism bear a heavy burden of care and often suffer from significant depression and anxiety symptoms, which is similar to the results of our study [27–29]. A study by Picardi et al. showed that parents of children and adolescents with autism suffer from high levels of objective and subjective burden. Mothers of these children reported more subjective burdens than fathers [29]. In justification of this finding, it can be said that the constant care of an autistic child is accompanied by mental stress, frustration and depression, which can affect the mental health of parents and caregivers responsible for supporting and caring for them [29]. Autistic children are a significant challenge for their parents. When parents are diagnosed with autism in their children, they face significant problems related to children’s symptoms and special and long-term care [30]. Parents have to meet the needs of themselves and their children simultaneously, which leads to increased pressure in all physical, emotional, social, and economic dimensions and changes in life following the provision of a caring role [31]. The studies indicate that the adverse effects of having a weak or disabled child cause tension and pressure on family members, especially the mother, because the mother is the first person to communicate directly with the child [32, 33]. Also, in explaining the intense burden of care for autistic children’s mothers, it can be said that the lack of support and social system that can provide therapeutic, medical, and occupational support to these children and their families is another serious problem in Iranian society [34].

Based on the results of this study, the average score of resilience in this research was 52.7, which indicates a moderate level of resilience. In Pastor et al.‘s study, the level of resilience of parents of autistic children was reported as moderate, which is in line with the results of our study. [35]. The average level of resilience of mothers of autistic children can be justified by the fact that the availability of a support system and the existence of support may be a means of adapting to the conditions in the role of caregiver, which leads to greater resilience [36]. Hesi et al. reported that a good support system is one of the factors related to resilience [37]. Also, as the nature of autism is a chronic disorder, it may reduce the perceived stress on caregivers over time because they may adapt to the behavioral patterns and fluctuations of the disease over the years and may become more flexible people as a result of getting used to it, and this is consistent with the findings of the present study [12].

The present study’s average score of coping styles was 92.4, which indicates an average level of coping styles. Also, among the subscales of problem-focused coping styles, positive re-evaluation with an average of 13.2, and Emotion-focused, avoidance, and avoidance styles got the highest scores with an average of 14.8. This result was in line with Hastings et al.‘s findings; They reported that the use of positive reappraisal of potentially traumatic and stressful events might be one of the only effective and available coping strategies for families with children with autism in severe situations [12]. Also, Rayan et al. stated that coping with positive re-evaluation by parents of autistic children reduces their stress [38]. In their study, Dunn et al. noted that the use of avoidance strategy among parents of autistic children is used more than other strategies, which can be a short-term stress reduction mechanism. However, avoiding the problem and not dealing directly with stressful events can be harmful and ultimately lead to family inconsistencies. Also, the avoidance coping style is related to increased depression and isolation [39]. Also, in the study of coping styles of parents of children with autism, Lai et al. found that these parents use avoidant coping more than parents with normal children [40]. Based on the present study’s findings, the average score for emotion-focused coping style was higher than the average score for problem-oriented coping style. There are conflicting results about common styles in families with autistic children. Wang et al. showed that avoidant coping was less common among parents of children with autism [41], while Lai et al. and Pisula et al. reported the opposite [40, 42]. These different findings in the existing studies show the need for more studies on coping strategies that mothers of children with autism specifically use.

The results obtained in this study aimed at determining the relationship between caregiving burden and coping styles in mothers of children with autism spectrum disorder showed no statistically significant relationship between caregiving burden and coping style. Based on previous studies, it was expected that mothers with more problem-focused coping styles report a lower burden of care, and mothers who use more emotion-focused coping styles report a higher burden of care. However, unlike many previous studies, the relationship between caregiving burden and problem-focused and emotion-focused coping styles was meaningless in this study [43–45]. In line with the present study, Stuart et al. conducted a research to investigate the factors affecting the burden of care after the diagnosis of autism spectrum disorder. The results indicated no relationship between caregiving burden and active coping styles [30]. Also, the findings of this study are consistent with the research of Osundina et al. They found no significant relationship between caregiving burden and coping style in caregivers of patients with mental disorders [46]. Smith et al. also stated that using problem-focused coping strategies minimally reduces mothers’ discomfort [47]. Also, the study by Hastings et al. showed that problem-focused coping is not related to stress and mental health of parents of autistic children [12].

In justifying the lack of relationship between coping styles and the burden of care, it can be said that coping styles are often used when a person is exposed to acute stress and uncontrollable stressful conditions [48], which means that according to The chronicity of the stress factor, mothers of autistic children have adapted to their long-term experience as caregivers. The use of coping styles does not affect their burden of care [12]. Also, through the cultural dimension, the results of this study can be justified as Iranian families accept and support their children despite any illness and have a sense of commitment towards the unchangeable situation [49]. In this regard, previous studies show that accepting and understanding the disease’s characteristics helps the family stay together even in the face of unpleasant problems when family caregivers can take the diagnosis and evaluate the critical situation positively. They can find different ways to adapt to their situation [50, 51]. Also, one of the possible explanations for the lack of relationship between coping styles and the burden of care is the small sample size in this study. More studies with a more complex design and a larger sample size may provide more information on this issue. Other research methods such as qualitative research may be necessary to understand how the mothers of these children cope with complex life conditions.

Also, the results obtained in this study to determine the relationship between caregiving burden and resilience in mothers of children with autism spectrum disorder showed an inverse and significant relationship between caregiving burden and resilience in mothers of children with an autism spectrum disorder. There are statistics, and the results obtained were similar to the results of previous studies [19, 52, 53]. Ozge et al.‘s study aimed to determine the relationship between caregiving burden and resilience and the quality of life of parents of children admitted to a rehabilitation center in Turkey, showed that there is a moderate negative correlation between caregiving burden and resilience, which was similar to the results of our study. 53). Also, Pipatananond et al. reported that resilience indicators reduce caregiver burden in family members of people with mental illness [54]. This relationship can be explained by reducing the amount of care burden and the stress caused by care, adaptability to adverse conditions increases, and as a result, resilience increases. In fact, a resilient person is characterized by self-efficacy. Such a person sees stress as a challenge and an opportunity, uses the support of others, accepts reality, has an extraordinary ability to adapt to important changes, and deeply believes that life is meaningful. In addition, a person with high resilience has a sense of humor, an action-focused approach, patience, and optimistic thoughts. All these characteristics help them bear the stress and burden of care effectively [24, 55, 56].

Finally, we can state that according to the findings of this research, mothers of autistic children shoulder a lot of burden in taking care of their children and have moderate adaptation to the existing conditions. Therefore, proper planning and policies are necessary to reduce the burden of care for these mothers. In addition, due to the importance of resilience, nursing interventions are essential to increase resilience. Also, longitudinal studies are needed to determine the long-term effects of resilience on the physical and psychological outcomes of people with autism and their caregivers.

### Limitations

The most important limitation of the current research in the method of collecting studies is the use of a self-assessment questionnaire with a large volume of questions, affecting the subject’s accuracy in answering all the questions correctly. Also, one of our limitations was the use of the available sampling method, which made it impossible to select the participants in the research randomly. Finally, the statistical population of this research is made up of 69 mothers of children with autism, so it is recommended to conduct this research on a large sample size so that the hypotheses can be rejected or confirmed more strongly.

## Conclusion

According to the results obtained in the present study and its comparison with other studies with similar goals, it was found that the mothers of autistic children experience intense caregiving and have a moderate level of resilience. There is a significant relationship between the burden of care and resilience, so paying more attention to the effective factors in increasing resilience is necessary. Based on this, nurses and policymakers of healthcare organizations can provide support, counseling, and training for these mothers to help them adapt to caring for children with autism and thus reduce their burden of care.

## Electronic supplementary material

Below is the link to the electronic supplementary material.

Supplementary Material 1

## Data Availability

Data is contained within the article.
