# Supplementary material for: Determining the relationship between over-care burden and coping styles, and resilience in mothers of children with autism spectrum disorder
Source: Ital J Pediatr. 2023 May 8;49:53. doi: 10.1186/s13052-023-01465-0 (PMC10169368; doi:10.1186/s13052-023-01465-0)
Supplement: Supplementary file 1 — Supplementary Material 1 [file 13052_2023_1465_MOESM1_ESM.docx]

| **Reviewer #1** | **Respond** |
| --- | --- |
| 1. Can we extrapolate these results with mothers from different geographic regions in the world. Does author think it will affect outcome? If yes how? | In my opinion, yes, although the sample of participants in this study does not allow for geographic generalization and the sample of each study is different from the sample of other studies. But the role of a mother everywhere in the world is a role born from love and compassion, and the role of a mother does not depend on geography. |
| 2. What is author's opinion about impact of children's age on care burden score ? Why it is not statistically significant in this study? | The results of this study clearly show that the mother's love has nothing to do with the child's age, and at any age the mother gives her compassionate and caring love. Although at a young age, due to the diagnosis of autism, more care is expected, and at older ages, the amount of care decreases.But with increasing age, the addition of providing more medical services and going to school, the mother's love increases to the same extent as before, and this role does not diminish. |
